# Epidemiologic, clinical, and laboratory findings of the COVID-19 in the current pandemic: systematic review and meta-analysis

**DOI:** 10.1186/s12879-020-05371-2

**Published:** 2020-08-31

**Authors:** Yewei Xie, Zaisheng Wang, Huipeng Liao, Gifty Marley, Dan Wu, Weiming Tang

**Affiliations:** 1 Dermatology Hospital of Southern Medical University, Guangzhou, China; 2 School of Public Health, Nanjing Medical University, Nanjing, China; 3 London School of Hygiene & Tropical Medicine, London, UK; 4 University of North Carolina Project-China, Guangzhou, China

**Keywords:** COVID-19, Epidemiology, Nature history, Clinical spectrum, Laboratory findings

## Abstract

**Background:**

The COVID-19 pandemic has affected the world deeply, with more than 14,000,000 people infected and nearly 600,000 deaths. This review aimed to summarize the epidemiologic traits, clinical spectrum, CT results and laboratory findings of the COVID-19 pandemic.

**Methods:**

We scoped for relevant literatures published during 1st December 2019 to 16th July 2020 based on three databases using English and Chinese languages. We reviewed and analyzed the relevant outcomes.

**Results:**

The COVID-19 pandemic was found to have a higher transmission rate compared to SARS and MERS and involved 4 stages of evolution. The basic reproduction number (R_0_) is 3.32 (95% CI:3.24–3.39), the incubation period was 5.24 days (95% CI:3.97–6.50, 5 studies) on average, and the average time for symptoms onset varied by countries. Common clinical spectrums identified included fever (38.1–39.0 °C), cough and fatigue, with Acute Respiratory Distress Syndrome (ARDS) being the most common complication reported. Body temperatures above 39.0 °C, dyspnea, and anorexia were more common symptoms in severe patients. Aged over 65 years old, having co-morbidities, and developing complications were the commonest high-risk factors associated with severe conditions. Leucopenia and lymphopenia were the most common signs of infection while liver and kidney damage were rare but may cause bad outcomes for patients. The bilateral, multifocal Ground-Glass Opacification (GGO) on peripheral, and the consolidative pulmonary opacity were the most frequent CT results and the tendency of mortality rates differed by region.

**Conclusions:**

We provided a bird’s-eye view of the COVID-19 during the current pandemic, which will help better understanding the key traits of the disease. The findings could be used for disease’s future research, control and prevention.

## Background

The emergence of COVID-19 has made it the first infectious disease pandemic in the twenty-first century. As of 20th July 2020, a total of 14,348,858 people got infected, and 603,691 were confirmed dead in 213 countries, territories, and areas globally [1]. While more than 30 countries had issued the highest level of response, the SARS-CoV-2 (pathogen of COVID-19) continues to spread in different regions around the world [2]. However, the key information on the virus epidemiology, clinical spectrum, and on the pathogen was delayed in response during the early outbreaks in many countries. To fill the research gaps mentioned above, this review article systematically summarizes global findings on the natural history, clinical spectrum, transmission patterns, laboratory findings, CT results, and risk factors of the COVID-19.

## Methods

### Search methods for identification of studies

We searched for publications in epidemiology and clinic domains of the COVID-19 broadly. The databases we searched were: CHKD v3.1 of the CNKI [in Chinese], PubMed, and medRxiv, by using such search terms as ‘COVID-19, SARS-CoV-2, and 2019 nCoV’ (See Additional file 1). The publication date was restricted from 1st Dec 2019 to 16th Jul 2020. Both English and Chinese were applied for the search. Only the full-text available human studies were eligible for selection. Like the real-time data, other data were obtained from health departments of multiple countries, global NGOs, and reputable media sources.

### Data collection and analysis

#### Selection of studies

**Fig. 1:**
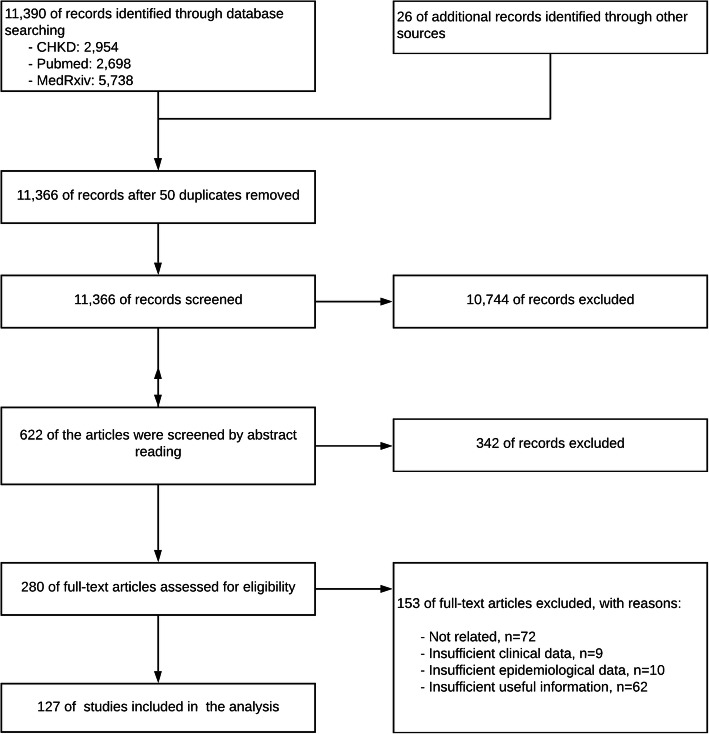
PRISM flow diagram

The searched records were firstly screened by reading titles and abstracts. Then, the rest records were screened again by full-text reading. If there were disagreements initially, the records then submitted to the whole team for further discussions. Besides, a PRISMA diagram was conducted to illustrate the entire flows of the review (Fig. 1).

#### Data extraction, management, and dealing with missing data

The data for the quantitative analysis was extracted and managed by using Microsoft Excel 2010 (Microsoft©, Redmond, WA, USA). The meta-analysis was performed by the R version 4.02 and RStudio (2020) [3]. The Cochrane Handbook for Systematic Reviews of Interventions suggested review authors collect missing data from investigators. Considered that using the imputation method to tackle the missing data problem could not reduce bias, we only analyse data available to us if we could not collect the missing data from the investigators [4].

#### Assessment of heterogeneity and reporting biases, and data synthesis

The heterogeneity of the included studies was assessed by using I^2^. The *P*-value was generated by Wald-type test and Likelihood-Ratio test. The overlapping Confidence Intervals (CIs) were displayed by the forest plots (See Additional file 4). We categorized and combined the data about epidemiologic traits, clinical spectrums, laboratory, and imageology findings in a narrative. Then we further analyze the data about common symptoms, reproduction number, and incubation period through meta-analysis. The quantitative outcomes were combined with the narrative of epidemiological and clinical findings.

## Results

We collected 11,366 records after removing duplications. After three batches of screening, 127 records were included in this review (See screening details in Fig. 1).

### Epidemiology

#### Demographic characteristics

In a China based study involving 55,924 COVID-19 patients, the majority of patients were aged 30–69 (77.8%) with only 2.4% of the patients being 18 years and below. The median age of the patients was 51 (ranged 2 days-100 years old) [5]. Similarly, in the United States, more than half of patients were aged between 20 and 64 years (65%), with only 5% of patients being under 19 years old. Older aged patients were more prone to getting infected compared to the young [6]. By gender, the male to female ratio of confirmed cases was 1.06:1.00 in China [7]. However, in South Korea and Iceland, the male population had a higher incidence rate than the female population [8, 9]. Males had twice the secondary attack rate than females [10].

#### Transmission stages

The COVID-19 transmission stages could be categorized into four temporal stages according to the chronological order of case reports. The first stage: people with exposure histories to Huanan Seafood Market (HSM) got infected [11]. Forty-one patients were found to be having SARS-like symptoms in December 2019, and the HSM was believed to be one of the origins of the virus. However, 13 of the 41 patients reported no prior exposure to the HSM thus indicating that the origin of the virus needed further investigation [12]. The second temporal stage is the transition from community transmissions to the outbreak in Wuhan [11]. The virus was mainly spread to multiple communities directly and indirectly by people with HSM exposure histories. The interpersonal transmissions and clustered transmissions formed community transmissions [11]. An earlier study showed that the proportion of patients with HSM exposure histories decreased from 55 to 8.6% within 22 days, indicating when people who did not have exposure histories to the HSM became infected [13, 14]. The third stage: the epidemic in China. At this stage, transmissions began to expand to communities outside Wuhan and the Hubei province as a whole [11]. On 26th Jan 2020, a study involving 62 COVID-19 patients outside Wuhan found that all the patients had been exposed to Wuhan, which demonstrated an established local transmission outside Wuhan [15]. The fourth temporal stage is the global pandemic. On 13th Jan 2020, the first case outside China was reported in Thailand [2]. On 30th Jan 2020, the WHO declared a Public Health Emergency of International Concern (PHEIC) [2]. It subsequently took about 51 days for transmission to escalate from the first reported case to the 10,000th reported case outside China. Globally, it took 16 days for the number of reported cases to increase from 10,000th cases to 100,000th cases, 21 days from 100,000th cases to 500,000th cases, only 6 days from 500,000th cases to 1,000,000th cases and 13 days from 1,000,000th cases to 2,000,000th cases [2].

#### Transmission routes

The main transmission route of this virus was by human-to-human spread, since only 1.18% patients among 1099 confirmed patients had history of direct contact with wild animals [16]. The vital transmission routes were through respiratory droplets and contact transmissions. There remains the possibility of aerosol transmission when exposed to high concentrations of aerosols for a long time in a relatively closed environment [17]. Mother-to-child transmission has been confirmed, whiles fecal-oral transmission was also considered possible but lacked direct evidence until now [18, 19]. Other suspected routes of transmission still needed further clarification.

#### Transmission patterns

Community transmission, nosocomial transmission, household transmission, and transmission in closed environments were four typical transmission patterns of the COVID-19.

Firstly, community transmission was considered to be an important pattern in COVID-19 spread [5]. In the Netherlands, community transmissions were found in the Noord-Brabant regions [20, 21]. In North America, community transmissions were reported in Winnipeg, Canada, and Eastern Idaho, United States [22, 23].

Secondly, the potential risk of transmission among medical personnel and through medical facilities was deemed high and thus extreme attention should be paid. Transmissions between patients and health workers were in higher proportions during the SARS outbreak, while transmission through medical facilities was higher in proportion during the MERS outbreak [24]. In Wuhan, the proportion of severely infected medical workers was higher than the national average [7]. In Italy, 2629 health workers were reported infected with the COVID-19 before 18th March and accounted for 8.3% of the total number of cases nationwide. The number however increased to 8358 by 30th March and represented 9% of the country’s total number of cases [25, 26]. In Spain, the number of diagnosed cases among medical workers increased to 5969 within 6 days and more than 12% of the country’s confirmed cases remained among medical workers until March 30th [25]. Update from another source reported an increase in the number of cases from 12 to 14% among Spain healthcare workers by 31st March and this was attributed to lack of medical supplies, such as masks and gowns. Other reasons accounting for these high infection rates among medical personnel varied according to different country’s circumstances. An Italy study pointed out hospitals as a potential hotspot for infection. Facilities and medical personnel turned into untested vectors and patients [27, 28]. In the US for example, the reasons that turned hospitals into infection hotspots included the overload of COVID-19 patients and inappropriate management against the pandemic in hospitals [29]. Similar to the US, 200 medical workers got infected in a county hospital in Romania due to inadequate hospital management. In Egypt, a serious wave of emigration by physicians for years led to patient overload for remaining medical workers and placed them at higher risk of infection through continuous exposure. The emigration wave was purportedly caused by low salary, undesirable working conditions, lack of legal protection, and shortage of medical supplies and equipment [30].

Thirdly, household transmission contributed to cluster infections and was the major transmission pattern observed in China. For instance, among 1836 reported cases in Guangdong and Sichuan Provinces, most cluster infections occurred in families (78–85%) [5]. The WHO in this regard issued a statement that household transmission highly occurred among medical workers’ families than health facility infection in China. Household transmission was also a significant pattern observed in South Korea and the US [2, 29]. The European Centre for Disease Prevention and Control (ECDC) had provided guidance for the control of household transmission in European countries [5, 31]. What made household transmission worse was that some groups (age < 18 and > 65) had high risk got infection within households than the general population [32]. So, children and elderly living with medical workers at a higher risk of getting than other populations.

Fourthly, transmissions in a closed environment besides the home should also be of a keen focus on the prevention and control of this outbreak. A Japanese health department reported that a closed environment could promote super-spreading events because the transmission of the SARS-CoV-2 in a closed environment was the same as large-scale transmission, such as the ski chalet-cluster infection in France and the church-hospital infection clusters in South Korea [33]. For example, outbreaks of the COVID-19 were observed in multiple prisons in China, the UK, and the US [7, 34, 35]. Cluster infections also happened on cruise ships, such as the Diamond Princess, Grand Princess, Golden Princess, Ruby Princess, Phoenix Reisen, MS Westerdam, and Punta Arenas [36]. Further studies are however required to identify and assess other potential transmission patterns for further prevention, especially since some cases were asymptomatic [37, 38]. In addition, patients who were considered cured and no longer needed quarantine still tested RT-PCR positive after 5 to 13 days [39].

#### Nature history

We systematically used the data of the incubation period and the reproduction numbers for meta-analysis (see details of selected studies on Additional file 2). The result suggested that the mean incubation period was 5.24 days (95% CI:3.97–6.50, 5 studies), and ranged from 3 to 7.4 days [40–44]. However, the incubation period in some special cases could be as long as 24 days [16]. The result also illustrated that the basic reproduction number (R_0_) of SARS-CoV-2 was 3.32 (95% CI:3.24–3.39, 14 studies) and varied between 0.6–6.47 [37, 42–53]. This finding suggested that the transmission ability of SARS-CoV-2 was stronger than SARS (3) and MERS (≤1) [54, 55]. Moreover, the median time from the first symptom to first hospital admission was 7 days with the median duration from illness development to severe symptoms development being: 5–8 days for dyspnea, 8–9 days for ARDS, 10.5 days for mechanical ventilation and ICU admission [6, 18]. For COVID-19 related deaths, the duration from the onset of symptoms to death averaged 9 days in China 5 and in Italy (median) [56], and 10 days in South Korea (median) [9].

#### Mortality and fatality

**Fig. 2:**
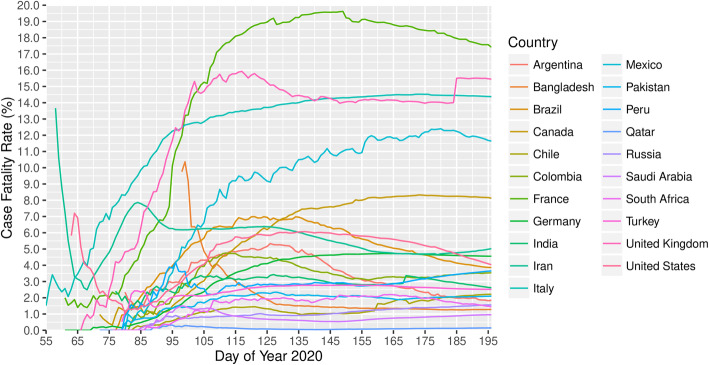
Case fatality rate of countries reported over 20,000 cases, 2020*. *Data was collected until 14 July 2020 (i.e. the 196th day of year 2020). The CFR of a country was not included on those dates when the country reported less than 100 cases, with the consideration that the CFR may not be reliable if the size of infected population was small

By 14th July 2020, 21 nations had reported over 100,000 COVID-19 cases in each of the countries, together contributed to 81.4% of the confirmed cases and 81.3% of death in the world1. The world case fatality rate (CFR) was 4.4% on 14th July; however, it was apparently different by country. One third of these 21 countries had a CFR of over 4.4%. France (17.4%), United Kingdom (15.5%), Italy (14.4%), and Mexico (11.6%) were the top four countries with over 10% CFR while Qatar (0.1%) and Saudi Arabia (1.0%) were the two countries with no more than 1% CFR. Most countries experienced an increase of CFR at first, and the number was then gradually becoming stable during the disease outbreak (Fig. 2). However, the CFR was high in Iran (13.7% on 27th February) and the United States (7.2% on 4th March) at first, experienced a sharp decrease to 2.5% on 8th March and 1.1% on 20th March, and rebounded to 5.0 and 4.0% on 14th July, respectively. Bangladesh was the only country that had high CFR of around 10% at the beginning and then continuously decreased until 1.3% on 14th July. As the pandemic outbreak continued, more surveillance is needed for the CFR of COVID-19 [57].

The mortality is higher among elderly, patients requiring intensive care unit admission and male. However, mortality rate among younger age group and patients with mildly disease is less. The US’s data indicated that patients younger than 19 had milder COVID-19 illness, with almost no hospitalizations or deaths reported [6]. Based on a worldwide data, the elderly (aged over 60) were at a high risk of developing into death [5, 6, 9, 56]. The mortality in ICU was extremely higher than Non-ICU patients, varied from 26 to 78% [58–61].

About the gender ratio, there is a seemingly unquestionable pattern that COVID-19 killed more men than women [62]. Unlike the less report in the research from China, South Korea or other Asia areas, the reports from Europe and American reflect the male gender is the risk factor for heavy illness. To figure out the general situation around the world, here we analyzed the data from 53 countries, compiled centrally and individually verified by authors against country-specific reports [63], shown that the case-fatality rate among male is about 35% higher than female (IR = 1.35, 95% Confidence interval: 1.35–1.35) (Table 1). The sex-disparity is consistent across age groups and regions. For example, the incidence rate ratios between male and female were 1.05 (95% CI: 1.05,1.05), 1.46 (95%vCI: 1.46,1.46), 1.46 (95% CI: 1.46, 1.46), 1.61 (95% CI: 1.61,1.61) and 1.64 (95% CI: 1.64, 1.64) among COVID-19 cases in Asia, Africa, North America, South America, and Europe, respectively (Table 1). By age groups, COVID-19 cases aged below 60 years old (IR = 2.74 95% CI: 2.66–2.82), have wider sex-disparity than those aged above 60 years old (IR = 1.83, 95% CI: 1.82–1.83), while deaths among cases younger than 40 years old are very rare.

**Table 1:** Gender based fatality rate ratio among COVID-19 cases in different region of the world (By July 9th, 2020)

Variables		Sex	Fatality rate ratio (95% CI)
**Region**	Global	Female	1.00
		Male	1.35 (1.35, 1.35)
	Asia	Female	1.00
		Male	1.05 (1.05,1.05)
	Africa	Female	1.00
		Male	1.46 (1.46,1.46)
	North America	Female	1.00
		Male	1.46 (1.46,1.46)
	South America	Female	1.00
		Male	1.61 (1.61,1.61)
	Europe	Female	1.00
		Male	1.64 (1.64, 1.64)
**Age**	< 60	Female	1.00
		Male	2.74 (2.66,2.82)
	60–69	Female	1.00
		Male	2.36 (2.33,2.40)
	70–79	Female	1.00
		Male	1.76 (1.75,1.76)
	80 and above	Female	1.00
		Male	1.91 (1.91,1.91)
	60 and above	Female	1.00
		Male	1.83 (1.82,1.83)

### Clinical spectrum

#### Common symptoms

Based on the data collected from selected articles [12, 15, 16, 58, 64–92] (details of selected articles were put in the Additional file 3), we conducted the meta-analysis using a random-effects model to identify the clinical feature of COVID-19. Fever (76.70, 95% CI: 64.86–85.44%) and cough (67.76, 95% CI: 60.06–74.61%) were the most common symptoms. Other common symptoms included: olfactory (44.40%), gustatory (38.16%), dyspnea (37.49%), fatigue (29.93%), sputum production (17.85%), sore throat (16.17%) and headache (15.49%). All the other data showed in Table 2. Besides, studies pointed out that most patients had more than one symptom [68, 70, 71]. Additionally, there were 20.9% of patients without viral pneumonia symptoms [16], which was opposite to previous studies [69, 70]. The asymptomatic cases varied from 21.9–49.5% [66, 68, 93, 94].

**Table 2:** Meta analyzed results of COVID-19 common clinical symptoms (By July 16th, 2020)

	Proportions	95% Confidence interval	Heterogeneity test, I^2^	Heterogeneity test, *P* Value	Number of studies
Fever	76.70%	64.86–85.44%	99.7%	0	21
Cough	67.76%	60.06–74.61%	99.2%	< 0.0001	21
Olfactory	40.80%	20.31–65.08%	99.4%	0	14
Gustatory	34.52%	18.83–54.50%	98.9%	< 0.0001	13
Dyspnea	37.49%	26.20–50.34%	99.6%	0	19
Fatigue	29.93%	14.22–52.39%	99.7%	< 0.0001	11
Sputum production	17.85%	9.25–31.65%	99.1%	< 0.0001	10
Sore throat	16.17%	10.05–24.98%	96.9%	< 0.0001	9
Headache	15.49%	7.83–28.33%	98.7%	< 0.0001	12

**Fig. 3:**
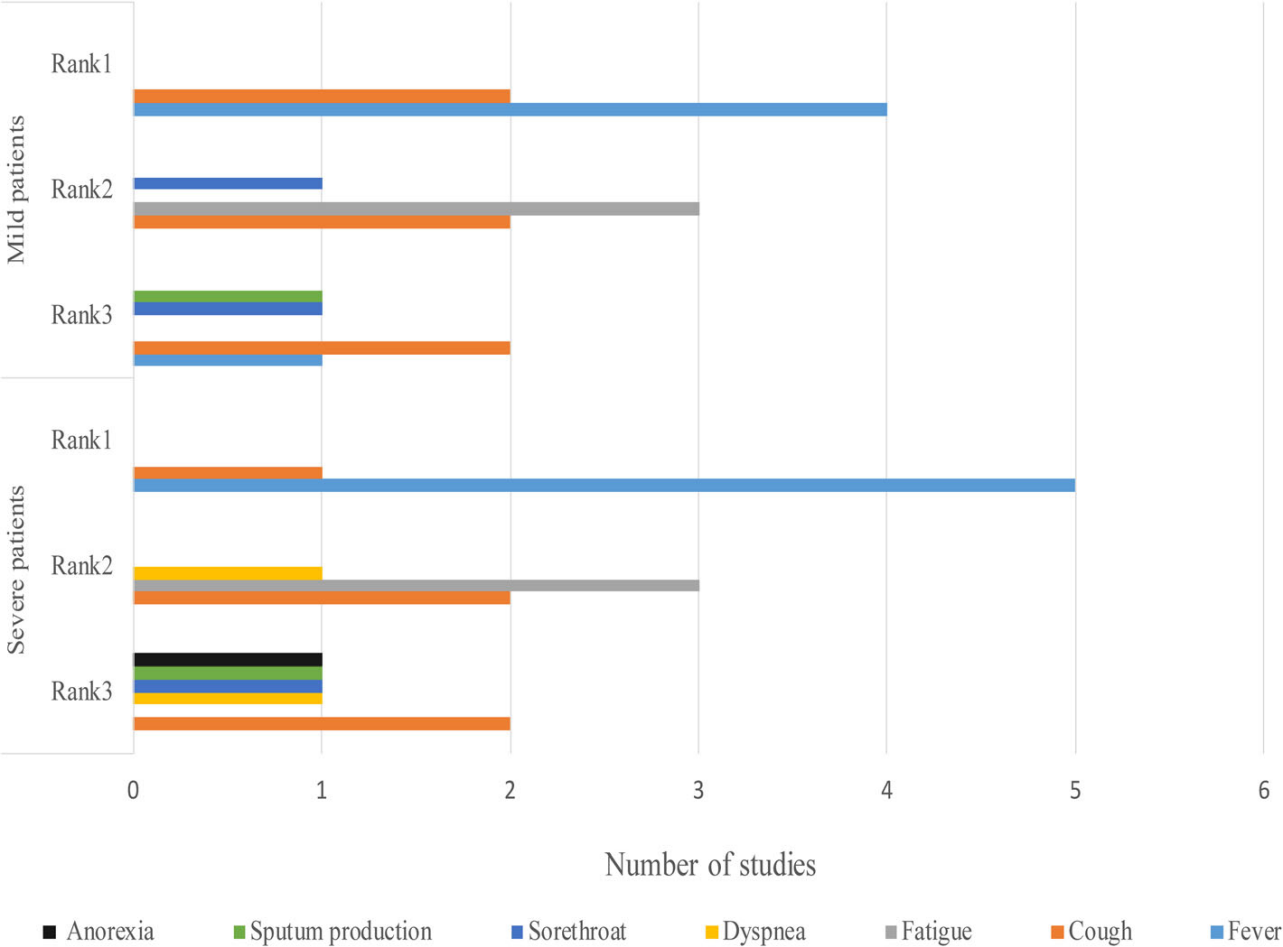
Comparison of top 3 symptoms among mild and severe patients with COVID-19, 2020*. *The X-axis means the number of symptoms reported by how many studies. The Y-axis means symptoms’ ranking in mild and severe patients. In this circumstance, rank means the order judged by the frequency of the symptoms reported among studies

The top 3 common symptoms among mild and severe patients are summarized and displayed in a figure (Fig. 3) [12, 16, 61, 70, 95–98]. Fever was found to be the most common symptom in all patients. In a study, 43.8% of patients had fever initially and the proportion increased to 87.9% following hospitalization [16]. The body temperatures of 44–47.1% of patients ranged between 38.1–39.0 °C. The higher body temperatures (above 39.0 °C), dyspnea and anorexia were more frequent among patients in severe conditions [16, 64, 98]. Cough and fatigue were more widely reported among mild and severe patients. Additionally, another study reported that dyspnea (76%) was the most common symptom among severe patients in the United States [99]. The proportion of patients who needed ICU care varied based on the local pandemic circumstances. For example, the WHO speculated that around 13.8% of patients were in severe conditions in China [5]. However, 23–32% of patients needed ICU care in Wuhan [64, 69, 70].

#### Common complications

Currently documented COVID-19 related complications include ARDS, arrhythmia, Septic shock, acute cardiac injury, myocarditis, acute coronary syndrome, cardiomyopathy, acute respiratory injury, and acute renal injury, etc. [58, 64, 67, 69, 70, 100]. The ARDS was the most common complication, among both mild and severe patients [58, 64, 67, 69, 70]. Most ICU patients had a higher risk of developing ARDS and having complications [12, 70]. The progress of some patients with ARDS to septic shock was fast and quickly evolved into multiple organ failure finally [69].

### Laboratory findings and CT scans

#### Laboratory findings

Among COVID – 19 patients, a decrease in leukocytes such as eosinophil and lymphocyte were commonly reported. This might be because the cytokine storm caused by the novel virus changes the peripheral of white blood cells and immune cells [12, 13, 15, 16, 69, 70]. Severe lymphopenia was also common among the dead patients [12, 61]. Myocardial zymogram abnormality was found in many patients. For instance, 76% of patients had an increase in lactate dehydrogenase, while 13% of patients had increases in creatine kinase [69]. The level of C-reactive protein was important to evaluate the infection [16]. Most patients were found to have a higher level of C- reactive protein (86%) and serum ferritin (63%) compared to the normal range [69]. The biomarkers related to liver and renal damage were found to be abnormal among COVID-19 patients. The abnormality of liver-related biomarkers was not widespread but yet still common in severe cases [12, 15, 16, 101]. Besides, although only 7% of patients showed renal biomarker abnormalities, renal damage might contribute to the final multi-organ failure and death outcome [102, 103].

The ICU patients showed higher levels of white blood cells, neutrophil counts, D-dimer, creatine kinase, and creatine with longer prothrombin times [12, 16, 70]. Compared to patients who survived, the patients who died had higher levels of D-dimer, high-sensitivity cardiac troponin I, serum ferritin, lactate dehydrogenase, IL-6, blood urea, creatinine, white blood cell counts and neutrophil counts. Severe lymphopenia was also common among dead patients [12, 61].

#### Computed tomography scan (CT scan) features

The Computed Tomography Scan (CT scan) was widely used for disease diagnosis, prognosis, and management during the COVID-19 [104]. The CT was found more sensitive for identifying SARS-CoV-2 patients than the RT-PCR assay (98% vs. 71%) in a study [105]. The CT evidence for confirming the highly suspected patients’ positive may precede the RT-PCR results [106, 107].

Most patients had GGO and the bilateral lung involvement [12, 69, 108–110]. One study found that bilateral lung involvement was more frequently shown in the intermediate course and late course, compared to the earlier clinical course [107]. The clinical course could be divided into four stages based on CT scan findings [110]. In the first stage (Pre-symptom), GGO, unilateral and multifocal were observed among most patients in this stage [107, 110]. In the second stage (symptoms ≤1 week), lesions soon developed into bilateral and diffused except for GGO. This stage was considered a period from transition to consolidation. A mixed pattern of transition and consolidation develops during this stage. In the third stage (symptoms 1–2 weeks), the GGO was still common and the consolidation pattern showed. Findings indicated an interstitial change, which was considered as the development of fibrosis. In the fourth stage (symptom 2–3 weeks), consolidation and mixed patterns were more common, and the GGO started to shrink [110], the consolidation was gradually absorbed among patients who recovered at last [111].

Among ICU patients, the bilateral multiple lobular and sub segmental areas of consolidation were considered typical findings [12]. Patients in severe condition showed diffuse lesions, with density increasing in both lungs. CT scans showed ‘white lung’ appearances, indicating the serious influence the infection has on patients’ lung functions [112].

### Risk factors

Being old (≥65 years old), male sex, having a higher BMI value (> 35 Kg/m^2^), having co-morbidities (e.g. hypertension, diabetes, cardiovascular and cerebrovascular diseases, etc.), and developing complications were vital risk factors for patients to develop severe conditions [59, 70, 78, 95, 96, 113, 114]. The cytokine storm, raised inflammatory markers, elevated cardiac troponins, the requirement of mechanical ventilation, and the requirement of intensive care unit stay predict the bad outcome of admission patients [61].

Findings from multiple studies showed that patients who are more than 65 years of age, with co-morbidities such as diabetes and heart diseases had a high mortality rate [61, 95, 115–117]. Late hospitalization and bacterial infections were also considered high risk factors for disease progression [69, 96, 116]. Smoking history could be a potential risk factor for developing severe conditions [69, 96]. People with underlying disorders were considered to be at a high risk of getting infected [5].

## Discussion

### Research gaps

Our review identified several research gaps. Firstly, large amounts of data from African were missing from this review. As the number of people in African suffering from malnutrition, anemia, malaria, HIV/AIDs and tuberculosis is high, a large “low immunity population” has been created which has made the control and prevention of COVID-19 in the region a challenge. The situation could be worsened by the limited health resources region [118] and hence, more African focused research is required to support Africa in fighting the epidemic.

Secondly, the proportion of asymptomatic patients is large but the current transmission ability by asymptomatic patients might be weak. However, further exploration of risks posed by the group is needed as limited studies exist on the subject matter [119]. Meanwhile, data on the distribution of asymptomatic patients in large-scale community groups is also lacking, prompting the need for large scale of active screening and testing to help identify them [93, 120]. This approach is however difficult and expensive for most countries to undertake as accurate strategies to identify asymptomatic currently are non-existent. Further research focus on asymptomatic patients is needed.

Third, a ‘super-spreader’ was defined as infected individuals who infected numerous others during the SARS outbreak. For example, a nephrotic hospitalized patient who infected 22 people was classified as a ‘super-spreader’ during the SARS in China. 19 in those 22 patients were medical workers who came in contact with the ‘super-spreader’. The incidence rate among the medical workers was 59.38% (19/32) in the nephrotic department [121]. In the COVID-19 era, the emergence of ‘super-spreaders’ were found in multiple places worldwide. A Saudi Arabian study linked the concept of ‘super-spreaders’ to ‘super spreading’ events noting that ‘super-spreaders’ might cause unexpected transmissions during the pilgrimage [122], as huge numbers of people gather. Reasons causing the super-spreading events might include: immune suppression, increased disease severity and viral load, asymptomatic individuals, and extensive social interactions [123]. However, the characteristics and features of how an individual becomes a super-spreader are still not clear [124]. Summarizing the features of the ‘super spreader’ concept, as well as their characteristics and role in transmissions, are needed in future disease control [125].

Fourth, it has been reported that some cured patients COVID-19 retested positive by PCR after being discharged and quarantined at home in multiple places [39, 126]. The reason for this phenomenon is still unclear and hence further investigations are required for future pandemic control [127].

### Limitations

There existed some limitations in this review. Firstly, this review was based on English and Chinese resources only. As the COVID-19 transformed from a regional outbreak to a global pandemic, comprehensive collection of the related information worldwide is needed. Secondly, the clinical spectrum presented in this review is based on general population only, and thus a further subgroup analyzes in future may help to figure out more on the entire picture of the COVID-19. For instance, although Kawasaki disease was found in children in the UK and Europe countries, other places did not report the gathering Kawasaki disease cases [128].

## Conclusions

The COVID-19 had a stronger transmission ability than SARS and MERS, timely intervention should be conducted to reduce the spread of the disease. The common symptoms included in this study could assist in identifying the potential patients. The summary of the common complications, lab findings, CT features and risk factors could help medical personnel better manage patients who may develop into severe conditions or death.

## Supplementary information

**Additional file 1.** Searching Terms.

**Additional file 2.** Detail of selected studies for meta-analysis (R0&Incubation period).

**Additional file 3.** Detail of selected studies for clinical symptoms’ meta-analysis.

**Additional file 4.** The forest plots of clinical symptoms’ meta-analysis.

## Data Availability

The key information and data generated and/or analyzed during this study were included in this article and/or its supplementary information files.

## Abbreviations

ARDS: Acute Respiratory Distress Syndrome
CFR: Case Fatality Rate
COVID-19: Coronavirus disease 2019
CT: Computed Tomography Scan
ECDC: The European Centre for Disease Prevention and Control
GGO: Ground-Glass Opacification
ICU: Intensive care unit
MERS: Middle East respiratory syndrome-related corona virus
PCR: Polymerase chain reaction
R_0_: Basic reproduction number
SARS: Severe acute respiratory syndrome
SARS-CoV-2: Severe acute respiratory syndrome corona virus 2
WHO: World Health Organization
