# Supplementary material for: Epidemiologic, clinical, and laboratory findings of the COVID-19 in the current pandemic: systematic review and meta-analysis
Source: BMC Infect Dis. 2020 Aug 31;20:640. doi: 10.1186/s12879-020-05371-2 (PMC7457225; doi:10.1186/s12879-020-05371-2)
Supplement: Supplementary file 1 — Additional file 1. Searching Terms. [file 12879_2020_5371_MOESM1_ESM.docx]

**Appendix**

**Appendix 1**

1. CHKD v3.1 of the CNKI (Journal) [in Chinese] (2,954 records)

((SU= Novel Coronavirus+Novel Coronavirus Pneumonia+Novel Coronavirus Pneumonia) AND (SU=Clinical+Epidemiological)) NOT (SU=Anatomy+Histology+Embryology+Biochemistry+Parasites+Pharmacology+Toxicology+Molecular Biology+Cell Biology+Psychiatry+Skin Disease+Venereal Disease+Gynecology and Obstetrics+Ear+Nose+Throat+Tumor+Physical Therapy+Sports+Anesthesia+Psychology+Traditional Chinese Medicine +Pharmacy)

1. PubMed (2,698 records)

| **Search** | **Query** | **Result** |
| --- | --- | --- |
| #3 | Search: #1 and #2 Filters: Full text, Journal Article, Humans, Chinese, English Sort by: Publication Date | 2698 |
| #2 | Search: (epidemi*[Title/Abstract]) OR (clinic*[Title/Abstract]) Filters: Full text, Journal Article, Humans, Chinese, English Sort by: Publication Date | 2674065 |
| #1 | Search: ((((COVID-19[Title/Abstract]) OR (SARS-CoV-2[Title/Abstract])) OR (2019nCoV[Title/Abstract])) OR (corona virus 2019[Title/Abstract])) AND (("2019/12/01"[Date - Publication] : "3000"[Date - Publication])) Filters: Full text, Journal Article, Humans, Chinese, English Sort by: Publication Date | 6388 |

1. medRxive (5,738 records)

for abstract or title "COVID-19, SARS-CoV-2, SARS2, 2019nCoV, epidemic, epidemiology, epidemiological, clinic, clinica" (match any words) and posted between "01 Dec, 2019 and 16 Jul, 2020"
