# Supplementary material for: Epidemiologic, clinical, and laboratory findings of the COVID-19 in the current pandemic: systematic review and meta-analysis
Source: BMC Infect Dis. 2020 Aug 31;20:640. doi: 10.1186/s12879-020-05371-2 (PMC7457225; doi:10.1186/s12879-020-05371-2)
Supplement: Supplementary file 2 — Additional file 2. Detail of selected studies for meta-analysis (R0&Incubation period). [file 12879_2020_5371_MOESM2_ESM.docx]

**Appendix 2** Detail of selected studies for pooling analysis

Table 3，Data used for R0 pooling analysis

| Author | R0(with 95 % CI) | Data Time period | Area | Sample size | Reference |
| --- | --- | --- | --- | --- | --- |
| Li J et al. | 4.38（95% CI: 3.63–5.13) | 10 January 2020–23 January 2020 | China | 530 | 44 |
| Li J et al. | 3.41(95% CI: 3.16-3.65) | 23 January 2020–8 February 2020 | China | 33167 |  |
| Li J et al. | 3.39(95% CI: 3.09–3.70) | 10 January 2020–8 February 2020 | China | 33697 |  |
| Wu JT et al. | 2.68(95% CrI 2·47–2·86) | 31 December 2019–25 January 2020 | Wuhan | 56 | 45 |
| Song Q et al. | 3.16(95% CI 2·90–3.43) | 15 January 2020–31 January 2020 | China | 11791 | 42 |
| Song Q et al. | 3.74(95% CI 3.63–3.87) | 15 January 2020–31 January 2020 | China | 11791 |  |
| Song Q et al. | 3.91(95% CI 3.71–4.11) | 15 January 2020–31 January 2020 | China | 11791 |  |
| Zhao S et al. | 2.24(95%CI: 1.96-2.55) | 10 January 2020–24 January 2020 | China | 1246 | 47 |
| Zhao S et al. | 3.58(95%CI: 2.89-4.39) | 10 January 2020–24 January 2020 | China | 1246 |  |
| Read JM et al. | 3.11(95%CI, 2.39–4.13) | 1 January 2020–22 January 2020 | Wuhan | 571 | 48 |
| Li Q et al. | 2.2（95% CI, 1.4 to 3.9） | By 22 January, 2020 | Wuhan | 425 | 13 |
| Liu T et al. | 4.5(95%CI: 4.4-4.6) | As of February 7, 2020 | China | 34598 | 50 |
| Liu T et al. | 4.4(95%CI: 4.3-4.6) | As of February 7, 2020 | Wuhan | 34598 |  |
| Liu T et al. | 0.6 (95%CI: 0.4-0.7) | Up to February 7, 2020 | Guangdong | 34598 |  |
| Tang B et al. | 6.47 (95% CI 5.71–7.23) | Until 22 January2020 | China | 571 | 51 |
| Liu T et al. | 2·90 (95%CI: 2·32-3·63) | As of January, 23 | Global | 839 | 52 |
| Liu T et al. | 2·92 (95%CI: 2·28-3·67) | As of January, 23 | Global | 839 |  |
| Zhang S et al. | 2.28 (95%2.06-2.52) | During the early stage experienced on the Diamond Princess cruise ship | the Diamond Princess cruise ship | 174 | 49 |

Table 4，Data used for Mean Incubation period pooling analysis

| Author | Mean Incubation period | Sample size | Reference |
| --- | --- | --- | --- |
| Linton NM et al. | 5（95%CI：2~14） | 315 | 40 |
| Backer JA et al. | 6.4（95% CI: 5.6 - 7.7) | 88 | 41 |
| Kucharski AJ et al. | 5.2(95% CI: 4.1-7.0) | 425 | 39 |
| Song Q et al. | 5.01(95% CI: 4.31-5.69) | 11791 | 42 |
| Wang P et al. | 7.4286 (95%CI 2-20) | 1212 | 43 |
