# Supplementary material for: Epidemiologic, clinical, and laboratory findings of the COVID-19 in the current pandemic: systematic review and meta-analysis
Source: BMC Infect Dis. 2020 Aug 31;20:640. doi: 10.1186/s12879-020-05371-2 (PMC7457225; doi:10.1186/s12879-020-05371-2)
Supplement: Supplementary file 3 — Additional file 3. Detail of selected studies for clinical symptoms’ meta-analysis. [file 12879_2020_5371_MOESM3_ESM.docx]

**Appendix 3** Detail of selected studies for clinical symptoms’ meta-analysis

Table 5, Data used for clinical symptoms meta-analysis

| Author | Area | N | Fever | Cough | Dyspnoea | Fatigue | Olfactory | Gustatory | Headache | Sore throat | Sputum production | Reference |
| --- | --- | --- | --- | --- | --- | --- | --- | --- | --- | --- | --- | --- |
| Hong KS et al. | South Korea | 98 | 62 | 58 | 32 | NA | NA | NA | NA | NA | 39 | 63 |
| Qasim M et al. | Japan | 1080 | 857 | 459 | 223 | 316 | NA | NA | NA | NA | NA | 64 |
| Shabrawishi M et al. | Saudi Arabia | 146 | 72 | 71 | 29 | 1 | NA | NA | 4 | 24 | 5 | 65 |
| Pongpirul WA et al. | Thailand | 193 | 121 | 95 | 25 | 30 | 11 | 8 | 25 | 54 | 41 | 66 |
| Almazeedi S et al. | Kuwait | 1098 | 59 | 318 | 30 | 38 | NA | NA | 70 | 128 | 24 | 67 |
| Chen N et al. | China | 99 | 82 | 81 | 31 | NA | NA | NA | 8 | 5 | NA | 68 |
| Wang D et al. | China | 138 | 136 | 82 | 43 | 96 | NA | NA | 9 | 24 | 37 | 69 |
| Xu X-W et al. | China | 62 | 48 | 50 | NA | 32 | NA | NA | 21 | NA | 35 | 14 |
| Guan W-j et al. | China | 1099 | 966 | 744 | 204 | 419 | NA | NA | 150 | 153 | 367 | 15 |
| Huang C et al. | China | 41 | 40 | 31 | 22 | 18 | NA | NA | 3  (n=38) | NA | 11  (n=39) | 11 |
| Docherty AB et al. | UK | 17452 | 12499 | 12896 | 12107 | NA | NA | NA | NA | NA | NA | 70 |
| Rojo JMC et al. | Spain | 6386 | 5440  (n=6385) | 4885 | 3666  (n=6366) | 2965  (n=6236) | 385  (n=6109) | NA | NA | NA | 1071 | 71 |
| Regina J et al. | Switzerland | 200 | 131 | 124 | 98 | 99 | NA | NA | NA | NA | NA | 72 |
| Boddington NL et al. | UK | 381 | 229 | 296 | 154 | 267 | NA | 168 | 216 | NA | NA | 73 |
| Colaneri M et al. | Italy | 44 | 40 | 15 | 10 | NA | NA | NA | NA | NA | NA | 74 |
| Argenziano MG et al. | New York （USA） | 1000 | 728 | 732 | 631 | NA | NA | NA | 101 | 84 | 83 | 57 |
| Ortiz-Brizuela E et al. | Mexico City (Mexico) | 309 | 245 | 264 | 124 | NA | NA | NA | 246 | 161 | NA | 75 |
| Shekhar R et al. | New Mexico (USA) | 42 | 40 | 43 | 40 | NA | NA | NA | NA | 3 | NA | 76 |
| Suleyman G et al. | Detroit (USA) | 463 | 315 | 347 | 282 | NA | NA | NA | 73 | NA | NA | 77 |
| Goyal P et al. | New York （USA） | 393 | 303 | 312 | 222 | NA | NA | NA | NA | NA | NA | 78 |
| de Souza WM et al. | Brazil | 1468 | 982 | 1040 | NA | NA | NA | NA | NA | NA | NA | 79 |
| Beltrán‐Corbellini Á et al. | Spain | 79 | NA | NA | NA | NA | 25 | 28 | NA | NA | NA | 80 |
| Be´ne´zit F et al. | France | 68 | NA | NA | NA | NA | 51 | 63 | NA | NA | NA | 81 |
| Giacomelli A et al. | Italy | 59 | NA | NA | NA | NA | 14 | 17 | NA | NA | NA | 82 |
| Kaye R et al. | US, UK, Italy | 237 | NA | NA | NA | NA | 172 | NA | NA | NA | NA | 83 |
| Klopfenstein T et al. | France | 114 | NA | NA | NA | NA | 54 | 46 | NA | NA | NA | 84 |
| Lechien JR et al. | Belgian, France, Belgium, Spain,Italy | 417 | NA | NA | NA | NA | 357 | 342 | NA | NA | NA | 85 |
| Mao L et al. | China | 214 | NA | NA | NA | NA | 11 | 12 | NA | NA | NA | 86 |
| Moein ST et al. | Iran | 60 | NA | NA | NA | NA | 59 | 14 | NA | NA | NA | 87 |
| Vaira LA et al. | Italy | 320 | NA | NA | NA | NA | 62 | 62 | NA | NA | NA | 88 |
| Yan CH et al. | US | 59 | NA | NA | NA | NA | 40 | 42 | NA | NA | NA | 89 |
| Song J et al. | China | 1172 | NA | NA | NA | NA | 134 | 241 | NA | NA | NA | 90 |
| Qiu C et al. | China, France, Germany | 394 | NA | NA | NA | NA | 254 | 100 | NA | NA | NA | 91 |
