# Supplementary figures and images for: Epidemiologic, clinical, and laboratory findings of the COVID-19 in the current pandemic: systematic review and meta-analysis
Source: BMC Infect Dis. 2020 Aug 31;20:640. doi: 10.1186/s12879-020-05371-2 (PMC7457225; doi:10.1186/s12879-020-05371-2)

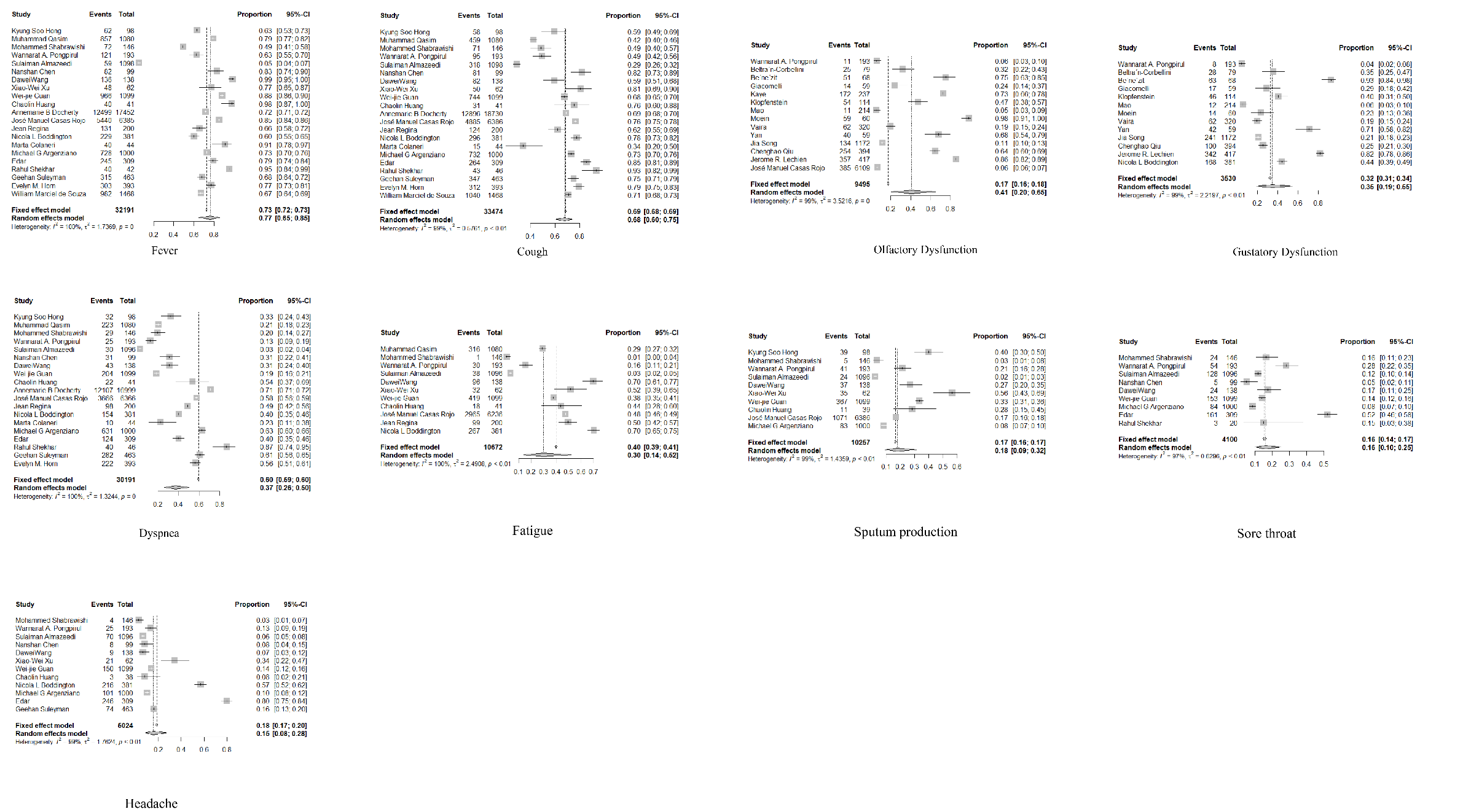
**Appendix 4** The forest plots of clinical symptoms’ meta-analysis

Supplement: Supplementary file 4 — Additional file 4. The forest plots of clinical symptoms’ meta-analysis. [file 12879_2020_5371_MOESM4_ESM.docx]
